# Editorial Department

**Published:** 1881-07

**Authors:** 


### The Bistoury.

ELMIRA, N. Y., JULY, 1881.

The BISTOURY is published Quarterly, upon the first of April, July, October and January, at Fifty Cents a year in advance.

### Editorial Department.

OUR DELAY, our readers have doubtless guessed, is attributable to our usual vacation in the woods. We did not return until July 9th, and all our editorial labor has been performed since that time. It would not have been resting to have written for the BISTOURY during the time we were camping out and trying to forget that any labor remained to be performed at home.

---

H.—Has, as we have heretofore hinted, an inquiring mind. He has been experimenting of late on the effect of certain articles of diet upon the human system. His last trial was a quart of cherries and a dish of ice cream as a luncheon, at bed-time. He has been, ever since, looking about for somebody to go with him to a convenient sanitary springs. He "don't feel well at all."

---

LARGE LAND HOLDER.—The largest land holder in the world is said to be Mr. Disston, of Philadelphia. He lately purchased two millions acres of land in Florida, and has paid the first installment thereon. Mr. Disston's father came to this country a poor man, but possessing a thorough knowledge of his trade, saw-making; before his death he had risen to the enviable position of the manufacturer of the best saws in the world.

---

GUITEAU'S PICTURE.—The assassin's phiz, as portrayed in many of the daily papers, is anything but a refined or intellectual one. He looks as though he was capable of doing just what he did. It is a matter for congratulation that the villainous attempt upon the President's life is thus robbed of any political significance. It would have been an everlasting disgrace to this great Republic to have had it otherwise. By the way, is it not time that some special punishment should be devised for these libertines who make attempts to destroy the lives of those at the heads of governments.

ANCESTRAL.—The baboon "Henry," at the zoological gardens, Phila., has been taught to wear clothes and to sit at the table and partake of his meals as would any of his more civilized descendants. It is said that he presents quite the appearance of a modern dandy.

---

PENALTIES.—Two brothers received a sentence the other day, for robbing a man of forty cents, of twelve years at hard labor. The assassin Guiteau will only get eight years for an attempt to murder the President. Is this "equal justice to all men?"

---

EFFECTIVE ADVERTISING.—What a magnificent advertising scheme the issuing of "bulletins," by the President's physicians, has become. It would have cost those doctors hundreds of thousands of dollars to have gotten their names and professional business before the world in the manner they have, had they adopted the usual methods of advertising.

---

WHY DEFEND?—Mr. H. L. Green, "Sec. of the Free Thinkers Association," invites Rev. Dr. Knox of this city, to "defend his faith" upon the platform before the Free Thinker's Convention which is to occur at Hornellsville this week. Just why the Docter should be called upon to "defend his faith" does not clearly appear. The Doctor and the community doubtless think it requires no defense.

---

PROBING FOR BULLETS.—There seems to exist an almost insane desire upon the part of many physicians to probe for a bullet, even when there is no certainty as to its locality. It is well enough to ascertain, by careful means, and disinfected probes, whether the ball is near the surface, and if so, to remove it. But to probe and punch blindly within the skull or abdomen, is vastly more injurious than the presence of any leaden bullet would be. The chap who thrust his finger into the wound of the President, performed a brilliant feat. In that act, he did almost as much harm as was already occasioned by the missle. A sweaty, dusty, perhaps infected finger, was a dangerous weapen in the hands of the ignoramus who wielded it.

---

BIG BASS.—Our friend C., who has been spending some time at the Thousand Islands enjoying the scenery and invigorating air and catching mammoth black bass on his fly rod, says that the tricks of the Heathen Chinee are no vainer than those of a three-pounder he encountered there. The fish finding himself snugly hooked, and having exhausted all his tricks of jumping out of water, sounding, and trying the strength of the flexible pole, came directly at the boat, jumped out of water against the side, knocked the hook out of his mouth and swam quietly away, giving his tail a peculiar tantalizing wag of derision and triumph. C. *went* for the heathen but was less successful than Bill Nye, and now claims advanced *peculiarity* for the black bass of the St. Lawrence.

---

SEA SIDE RESORTS.—It has come to pass that a short stay at the sea side is considered a necessity with every family laying claim to "position in society." Some good, sensible folks go there as a means of restoring health, or for rest from business too vigorously followed. To the latter class, a word of caution is offered. Such places, generally, are not calculated to give you health or rest. They are usually situated upon a level sandy beach, with the vilest of unsanitary surroundings. As for instance: We know of a fashionable resort of this kind on the New Jersey coast, visited yearly by one hundred and fifty thousand people. There are eight sewers that run through the town, emptying their garbage into an open ditch that in turn discharges its contents into a bay just outside the town. When the wind is favorable, the stench from this vile cesspool is abominable and we need not add sickening. The high living, fashionable dressing, late dancing and constant excitement—to say nothing of the disaster to one's purse, is not calculated to add much to health, restfulness or contentment of a sick or weary person.

We have so often given our views upon how to spend a summer's vacation that it would be superfluous to reprint them. It seems perfectly plain to us, that fashionable resorts of all kinds, wherever located, are *not* conducive to health or comfort.

---

C. M. BEECHER.—The readers of the leading newspaper in the Southern Tier—*The Elmira Daily Advertiser*—have noticed the signal ability with which that excellent journal has been edited during the past year. It was thought, when Mr. Fairman relinquished the editorial management to superintend the Insurance Department at Albany, the *Advertiser* would lose its prestige as a vigorously conducted Republican newspaper. It soon became manifest, however, that Mr. Beecher—to whom editorial control had been given—was the equal of his predecessor as a vigorous and voluminous writer. In no sense did the *Advertiser* lose its hold as an influential and powerful exponent of the principles and aims of the political party of which it was the avowed and recognized organ in this portion of the State. Although Mr. Beecher had been connected with and had edited various newspapers for a long series of years, he had never had an opportunity of exhibiting his great talent as an editor until he assumed editorial control of the *Advertiser*. He conducted that journal through a complicated and exasperating political muddle with a skill and excellent judgment that would have done honor to the veteran editor, Mr. Fairman, himself. No political editorials were more widely copied and commented upon than Mr. Beecher's, and none more sound and wise in their recommendations. We are sorry that we did not have an opportunity to pen these words of commendation during Mr. Beecher's life-time, for now he is dead and past reaping any gratification from what his fellows may say of him. He died suddenly, on the morning of July 8th, from a ruptured aneurism, having but just retired after completing his editorials for the morning paper. His death, we doubt not, was hastened by the severe mental and physical exertion to which his onerous duties subjected him. He worked, too, without knowing that his efforts were successful, and under a constant fear that he was not equal to the task imposed upon him. This feeling was engendered of an humble, sensitive nature that esteemed his co-laborers better than himself, and his own works inferior to theirs. Every one who came in contact with Charley Beecher—for so he was affectionately called—loved him for his modesty, his generous nature and his gentle, pleasing manner. Had he lived, it seems manifest that he would have speedily become one of the ablest and most distinguished editors in the land. We regret his death, sympathize with his bereaved family, and believe that his blameless and busy life will be acceptable to the Divine Master.

---

DR. BAXTER AND DR. BLISS.—When the President was so grievously wounded, it seems that Secretary Lincoln, without consulting any one, made haste to summon Dr. Bliss to the aid of the suffering man. The Doctor immediately took charge of the case, and suggested that the physicians who came at the call of various parties anxious to secure what they deemed the best advice, should be discharged, save only Dr. J. J. Woodward, Surgeon General Barnes, and Dr. Reyburn. These four medical gentlemen have ever since remained in charge of the President's case, and have conducted it with skill and rare good judgment. Dr. Bliss is installed as the chief attending physician, the other medical gentlemen being consulting physicians.

Hitherto, Dr. J. H. Baxter, Chief Medical Purveyor of the Army, has been recognized as the President's physician, having attended him in all former ailments, up to the present lamentable catastrophe; but Dr. Baxter, having left the city late on the Friday night preceding the shooting of the President, was unable to reach him until Sunday morning, at which time he promptly presented himself at the White House, and sought to see his patient and friend. He courteously sought the attending physician, Dr. Bliss, and asked permission, very justly, to see the President, when he was flatly refused by Dr. Bliss, and, it would seem at this distance, most outrageously treated. The exact language indulged in at this interview, and which has been variously stated by the press, is set forth in the following circular letter, which Dr. Baxter has issued to the medical fraternity:

WASHINGTON, D. C., July 7th, 1881.

DEAR DOCTOR:—I think it due you, as my friend, that you should know precisely my status in the President's case.

I have been President Garfield's family physician for the past five or six years, and since his advent to the White House have continued to treat him professionally.

Mrs. Garfield prefers homœpathic treatment, and in her recent illness I had no professional connection with her case.

At the time the President was shot, I was absent, having left the city twelve hours previously, to spend a few days with a friend near Williamsport, Pa., but on receipt of the news of the shooting, I returned by first express train, reaching Washington Sunday, July 3d, at 9 A. M.

I went directly from the depot to the White House, and finding Dr. D. W. Bliss, said to him, "Doctor I have come to ask you to take me in to see the President." He replied, "Well, I don't see the necessity of your seeing the President; I wish to keep him quiet." Somewhat astonished at his reply, I said, "I make the request as the President's physician; I have for years been his physician." "Yes," replied Dr. Bliss, "I know your game; you wish to sneak up here and take this case out of my hands." I said, "I wish nothing, Dr. Bliss, except what I am entitled to. If the President prefers that you should take charge of his case I haven't a word to say." "Well," said Dr. Bliss, "you just try it on. I tell you that you can't do it. I know how you are sneaking around to prescribe for those who have influence and will lobby for you." "That is a lie," I replied. Whereupon he sprang to his feet, and his son coming across the room placed his hand on my shoulder and said, "I think I have something to say about this." The impropriety of having any disturbance in a room next to that in which the President lay so grievously wounded at once came to my mind, and taking my hat, I left the room, and have not since attempted to visit the President. I believe, as do other members of the profession in this city, that the treatment I received was discourteous in the extreme, and that in making the request I was fully justified by the Code of Medical Ethics of the American Medical Association.

I had no desire nor intention to dispense with the medical services of Dr. Bliss in the case, but thought as I was the physician of the President I had a right to see him and take part in his treatment.

Sincerely your friend,

J. H. BAXTER.

From this, it clearly appears that Dr. Bliss not only violated the medical Code of Ethics, by which all honorable physicians are supposed to be governed, but conducted himself more like a bully than a refined medical gentleman. His clear duty, as specified in the Code of Ethics referred to, was, to not only promptly admit the recognized physician of the President to his presence, but to at least *offer* to turn the patient over to Dr. Baxter. In not doing so, he committed a grievous sin against the Ethics governing medical men in all such cases, and against the good manners and gentlemanly deportment recognized of all men.

Dr. Baxter did a courageous and manly act to withdraw, and so prevent a disturbance that would have greatly annoyed and distressed the President. Physicians throughout the country, when they come to learn these facts in the case, will not be slow to condemn Dr. Bliss' inexcusable action toward Dr. Baxter, and to applaud the latter for retaining his temper and not creating a scene in the apartment next the distinguished invalid.

Another phase of the question seems very curious to us, and that is, that Dr. Bliss should find such distinguished physicians as Drs Woodward, Hamilton and Agnew willing to consult with him, when it is remembered that he was dropped from the Medical Society at Washington because of his connection with the *cunderango* swindle,—a medicine that he sought to patent as a cure for cancer. How is it that these great physicians dare violate the precious "code" in this manner, when lesser lights doubtless would suffer discipline at the hands of their respective societies?

---

THE NATIONAL CALAMITY.—The nation mourns, fortunately not the death, but the attempted murder of her Chief Ruler. The hourly bulletins from Washington are eagerly scanned by crowds of horror-stricken patriots; all party strifes sink into insignificance in the presence of the National Calamity. As the hope of the people is strengthened by favorable reports from the surgeons in attendance upon the President, the causes and results begin to occupy the attention of men of thought.

Let us look into the causes which have tended to the commission of this crime. Given a fanatical fiend, ready for any deed to gratify revenge for a supposed wrong, or to work out a damnable notoriety by some hideous crime, how little is then necessary to induce such an attempt as that of Guiteau ?

Without commenting upon what faults of early training made this monster; without tracing the one folly of an otherwise honorable, upright parent, who consigned this son to a residence and association with a community whose peculiar views nurtured the germs of reckless fanaticism and crime, which that father too late realized and could not control; without touching upon the warning to parents and the importance to the nation of the proper training of youth, let us stop and seek for the cause which turned this fiend's evil into a national channel.

We are told that he had sought for public office and been refused. He says himself that he was entitled to the place he sought, because he worked side by side with others, successful party leaders. His words intimated even more, that President Garfield removed, he would be taken care of, and he now prays in prison for two things: the President's death, and to be protected from the incensed public.

General Jackson introduced into the politics of this country a curse, "To the victors belong the spoils," which has become a stench in the nostrils of the nation, and out of which has grown evils that have now culminated in this great crime. The advocates of this system are to-day in a painful position. Of course no one having any proper realization of the character of the men prominent just at this time as defenders of official patronage can suspect for one instant that they would countenance such a deed, but they must see themselves that to the system they defend, this crime, and much of the bribery and corruption of politics, is due.

General Jackson began with the dismissal of men from the higher offices to make room for party friends Since then the custom has reached down to the lowest employees of the Government, and out of it has sprung a division of our country into principalities, little and great, where the petty prince accepts the power and dispenses the patronage, regardless of the duties of the office or the fitness of the incumbent, so that his henchman has the place he asks for.

From a business standpoint let us consider this briefly: There is no office in the gift of the Government which does not require some special attribute or preparation to fill properly, and there is no position that is not better filled by a competent man, who has made himself fully acquainted with its duties.

Ask any business man if he would not prefer a clerk who is capable and familiar with his business to a new one, who has it all to learn.

Ask those who have business at the custom-houses and Government offices in our large cities as to the effect of frequent changes of clerks, the turning out of men who have just become familiar with the duties of the office and installing new ones, ignorant of all business except the management of ward politics.

Ask those who have been called to fill the high positions in our Government, of the disgraceful scrambles for office, the terrible ordeal through which a President or Secretary has to pass in the early months of his term, from the persistent petitions of these hungry office-hunters. It is officially stated that there have been filed, since President Garfield's inauguration, one million petitions for office! Imagine the personal applications of these petitioners and the introductory letters of their friends!

It would be needless to enter into a statement of the moral rottenness of political campaigns. We all know more or less of it—the less the better; but it would be easy to show that if, in a Presidential election, we merely voted for a President, and not for a chance for a million of office-seekers to get office, or a half a million to be turned out, the people would vie with each other, not in lying and cheating, but in an effort to secure the best man for the Nation's President.

Those of the public who are not seeking office are fast becoming advocates for a permanency in the minor offices, and perhaps a lengthening of terms in the higher ones.

Let us not forget, in writing of the results so far reached, of this great crime, to note the depth of national feeling manifested for President Garfield and his family. It has been almost wonderful. A nation's love seems to have welled up toward the man, independent of the office. Out of the depth of the shock, and amidst the terror of a great national calamity, the man, the mother, the wife, the children are sympathized with and cared for.

> ——— "Thro' plots and counterplots,
> Thro' gain and loss—thro' glory and disgrace—
> Along the plains, where passionate Discord rears
> Eternal Babel—still the holy stream
> Of human happiness glides on."

---

HOW TO REST.—All men will not find rest by the same means. A man, weary from manual labor, will not require the same sort of recreation as one who has become weary in office or counting room. A mechanic or laborer requires absolute rest of body when he seeks to escape from weariness, while a professional man, weary in brain and nervous system, requires a different condition of affairs, entirely. It would be no rest for a brain-weary man to go to bed and seek sleep and repose—he could not secure it there. He needs manual labor, fresh air, isolation from business and business men. Hence, a sensible man, really in need of such rest, will not hie him to a fashionable watering place, but rather seeks the retirement of the woods, where he can walk among the birds and flowers, paddle on the lake or pond, and amuse himself with rod and gun, while he drinks in the pure mountain air. All this by way of introduction to our tenth annual camping out expedition to "Bodines," that lovely retreat amid the mountains and rippling brooks that so often has afforded us much enjoyment and healthful rest.

With our good friend H., who has never failed us in our annual excursion to the woods, and George in charge of the two great chests containing the necessary cooking utensils and abundant supplies for the table, and our tents, bedding and fishing tackle, we departed on an early train for Bodines, on the Lycoming, on June 14th, reaching our destination by a little after eleven o'clock the same morning. By diligent endeavor we had our camping equipments transported to the grounds, and one tent securely in place before the usual and expected thunder storm overtook us. Some Philadelphians had occupied our grounds after we had left them the year before, and had used our tent poles—which we had carefully stacked against an old beech tree—for fire-wood. This necessitated a vigorous chopping of new poles and crotches, so that by the time we were sheltered, we were prepared to rest from our "rest" and enjoy the refreshing shower. H., who is no admirer of thunder—though he cares but little for the lightning—thought it dryer outside, under the beech, yet we strongly suspect this preference was developed from an idea of his that lightning (or thunder, we really do not know which,) was never known to strike a beech tree. The shower had scarcely passed over before the farm wagon arrived with our bed ticks, filled with hay. Hitherto, we had been able to secure oat straw, but upon this expedition it was nowhere to be found; and, we desire right here, to chronicle our disapproval of that sort of a commodity as a stuffing for bed ticks; it would do quite as well in a turkey and afford as little pleasure. Hard! Cobble stones would have been infinately better and a deal more elastic! By spreading a cotton filled quilt over it, however, we managed to secure a tolerably comfortable bed which the artist of the expedition declared was eiderdown in comparison with the hay-stuffed-contrivance. By dark, we had our canopy spread above our dining table, our ice house filled with ice from a neighboring farm house, our meats, butter and milk suitably arranged therein, when we were prepared for George's announcement of a supper of broiled steak, boiled potatoes, bread with good fresh butter (supplied by mother Bodine,) to which we sat down tired and hungry. A smoke and a chat about a blazing camp fire closed the day, when we retired to our beds and were lulled to sleep by the musical ripple of the cascade close at hand, accompanied by the song of the whippoorwill and the occasional hoot of some lonely old owl, that seemed to have lost himself somewhere far back in the dense forest.

In the morning, bright and early, we were all astir, for who could sleep with the bright sunlight shining through the tent, the merry birds making the fresh morning air musical without? A breakfast of trout—for H. had taken care the evening before that they should be supplied; coffee of fragrant aroma, and flapjacks that not only satisfied the palate, but afforded amusement as well (in flopping them up and catching them in the process of baking,) and then the preparations for invading the streams in pursuit of trout, occupied the early morning hours. The sport of trout fishing, the exhilarating and delightful exercise attending the occupation, need not be dwelt upon. The pleasures of angling are known to almost all men, who, at some time in their lives, have sought and found this inspiring and satisfying recreation. For weeks, in the cool and refreshing morning air, before the sun had obtained a position high enough to render his rays oppressive, and while the dew was yet glistening and sparkling upon the bright green grass and the birds making the mountains ring with their cheery songs, we were abroad upon the mountain streams, casting the fly for, and landing, many a sprightly trout. In the heat of the day, we laid off in our hammocks, or stretched ourselves upon a robe under the great branches of a far-reaching beech, speculated upon themes suitable to our surroundings, and laid plans for further angling excursions. During these days, friends called upon us, loitered about our camp, praised our surroundings and brought us news of the outside world. Ladies, with huge lunch baskets, invaded our forest home, climbed the mountain paths, collecting wild flowers and ferns with which to ornament their more civilized apartments at home. Our children, with their mother, occupied the family tent under the great elm, the artist another upon a grassy bank overlooking a beautiful pool, George, a tent to the front and between them both, while the spare tent, for visitors, stood in front of them all, facing the great pond upon which our boats were resting. These white edifices, with the dining room canopy and the cooking department, made quite a little village, that presented much the appearance of a "camp meeting," or perhaps, the grounds of a first-class circus company. The boys, with an eye to the ridiculous, had laid out the village in streets, giving them grotesque names, and had also labeled the different tents in the same manner. The family tent bore the name of "Palais Royale." The artist's, "Reformed Pirate's Retreat," with a well-painted picture of a pirate, in full uniform, on the fly. The guests' apartment bore the label, "Bridle Chamber—Halters Inside." Upon the side of the cook's tent were emblazoned a skull and cross-bones, with the legend, in bold letters, "Down with Victuals!" A camping party would scarcely be complete without a bevy of children to amuse and annoy the older members. Their resources for play, even with such facilities as nature affords, are inexhaustible and exceedingly amusing to a quiet looker-on, comfortably poised, pipe in mouth, under the shadow of a convenient tree. A fragment of knotty limb is readily converted into a doll by the little girls, by tying a piece of ochre-colored canvass (that once surrounded a ham) about one end to locate the neck and that the head might be distinguished from the trunk. This baby is rocked to sleep and talked to in the hammock as would be a live and kicking baby by a real mother. The boys build forts of boulders, laboring for hours to erect a wall that they will demolish in a second by a well-directed stone, that answers the purpose of a cannon ball. They will capture newts and frogs from the pond, harness them with strings, and then convert them into quite obedient teams. They investigate woodchuck and kingfishers' nests, and even dare to plug the entrance to a hornet's castle, and carry it triumphantly to camp to stampede the lazy loungers there to their tents, as the plug and the hornets come out at the same time. "What of it?" cried Fritz, when upbraided for this performance, as he stood with a diabolical grin upon his swollen face, his bare legs wrapped in bandages soaked in saleratus water. "Didn't yer say yer wanted exercise?" "Goodness! I haven't seen any of ye run so lively afore!"

Another prank of Fritz's was, when forbidden to sit about the camp fire one evening as a punishment for some disobedience of orders, to capture thousands of lightning-bugs and set them free within the family tent. And there he was found at bed-time, kicking up his heels, the tent ablaze with phosphorescent light, enjoying as he declared, his own "camp fire." And so the younger mem bers of the expedition enjoyed themselves, never lacking for devices to while away the pleasant hours in camp. The ladies busied themselves with their fancy work and books; the men with angling, constructing rustic work about the camp grounds, sketching and painting, until the full time allotted for the vacation had expired and we were regretfully compelled to turn our faces homeward. Not only have we had a joyful, restful and healthful time, but we have succeeded in convincing our many visitors from the city that no watering place in the land is comparable, for healthfulness and comfort, to camp life in the woods.

